# Co-expression of CD147 (EMMPRIN), CD44v3-10, MDR1 and monocarboxylate transporters is associated with prostate cancer drug resistance and progression

**DOI:** 10.1038/sj.bjc.6605839

**Published:** 2010-08-24

**Authors:** J Hao, H Chen, M C Madigan, P J Cozzi, J Beretov, W Xiao, W J Delprado, P J Russell, Y Li

**Affiliations:** 1 Faculty of Medicine, UNSW, Kensington NSW 2052, Australia; 2 Cancer Care Centre, St George Hospital, Gray St Kogarah, Kogarah NSW 2217, Australia; 3 School of Optometry and Vision Science, UNSW, Kensington NSW 2052, Australia; 4 Department of Surgery, St George Hospital, Kogarah, NSW 2217 Australia; 5 Department of Anatomical Pathology, St George Hospital, Kogarah, NSW 2217, Australia; 6 Douglass Hanly Moir Pathology, North Ryde, NSW 2113 Australia; 7 Oncology Research Centre, Prince of Wales Clinical School, UNSW Randwick, NSW 2031, Australia; 8 Australian Prostate Cancer Research Centre-Queensland, Institute of Health and Biomedical Innovation, Queensland University of Technology, 60 Musk Avenue, Kelvin Grove, QLD 4059, Australia

**Keywords:** prostate cancer, CD147, CD44v3-10, monocarboxylate transporters, multidrug resistance, metastasis

## Abstract

**Background:**

The aim of this study is to seek an association between markers of metastatic potential, drug resistance-related protein and monocarboxylate transporters in prostate cancer (CaP).

**Methods:**

We evaluated the expression of invasive markers (CD147, CD44v3-10), drug-resistance protein (MDR1) and monocarboxylate transporters (MCT1 and MCT4) in CaP metastatic cell lines and CaP tissue microarrays (*n*=140) by immunostaining. The co-expression of CD147 and CD44v3-10 with that of MDR1, MCT1 and MCT4 in CaP cell lines was evaluated using confocal microscopy. The relationship between the expression of CD147 and CD44v3-10 and the sensitivity (IC_50_) to docetaxel in CaP cell lines was assessed using MTT assay. The relationship between expression of CD44v3-10, MDR1 and MCT4 and various clinicopathological CaP progression parameters was examined.

**Results:**

CD147 and CD44v3-10 were co-expressed with MDR1, MCT1 and MCT4 in primary and metastatic CaP cells. Both CD147 and CD44v3-10 expression levels were inversely related to docetaxel sensitivity (IC_50_) in metastatic CaP cell lines. Overexpression of CD44v3-10, MDR1 and MCT4 was found in most primary CaP tissues, and was significantly associated with CaP progression.

**Conclusions:**

Our results suggest that the overexpression of CD147, CD44v3-10, MDR1 and MCT4 is associated with CaP progression. Expression of both CD147 and CD44v3-10 is correlated with drug resistance during CaP metastasis and could be a useful potential therapeutic target in advanced disease.

Prostate cancer (CaP) remains the most common cancer and the second leading cause of death from cancer in males in the United States (Jemal *et al*, 2009). Although early-stage CaP can be controlled using conventional therapies, multidrug resistance (MDR) and tumour metastasis remain the main causes of treatment failure and mortality in CaP patients. The majority of deaths in CaP result from progression to androgen-independent disease (Valdespino *et al*, 2007). For androgen-independent CaP, chemotherapy is the standard treatment option for palliation of symptoms associated with the disease. However, the drug-resistant nature of CaP minimises therapeutic efficacy, and consequently, most patients die within 12 months. The relationship between tumour metastasis and MDR is not fully defined in CaP, although indirect evidence in advanced disease suggests a functional link between these processes

CD147 (EMMPRIN, extracellular matrix metalloproteinase inducer protein) is a multifunctional glycoprotein that can modify the tumour microenvironment by activating proteinases, inducing angiogenic factors in both tumour and stromal cells, and regulating growth and survival of anchorage-independent tumour cells (micrometastases) and MDR expression (Yan *et al*, 2005). CD147 is highly expressed on the surface of various tumours, including CaP, and is associated with cancer progression (Riethdorf *et al*, 2006). Transcriptome analysis and comparative genomic hybridisation of individual tumour cells isolated from the bone marrow of patients with CaP have shown that CD147 is the most frequently expressed protein in primary tumours and micrometastases (Klein *et al*, 2002). Moreover, CD147 expression is considered a significant prognostic factor in human CaP (Zhong *et al*, 2008). Our recent results show that high-level CD147 expression is significantly correlated with CaP progression to high-grade disease, and is associated with the expression of MMPs in both tumour and stromal cells, including fibroblasts and endothelial cells (Madigan *et al*, 2008).

CD44 is a multifunctional protein involved in cell adhesion, migration and drug resistance. Alternative splicing of the CD44 gene produces many CD44 isoforms or variants (CD44v), some of which form the invariant extracellular domain of standard CD44 (CD44s). CD44s is expressed in the majority of normal basal prostate cells; however, CD44v expression is reported in CaP (Harrison *et al*, 2006). The role of CD44 in CaP development and progression is controversial, with studies showing both tumour-promoting and tumour-inhibiting effects (Gao *et al*, 1998; Omara-Opyene *et al*, 2004). Clearly the involvement of CD44 and its variants in CaP progression and metastasis is complex.

A major mechanism for drug resistance in cancer is through energy-dependent efflux pumps that reduce intracellular drug accumulation. One of these, which is well characterised, is MDR1/P-glycoprotein (P-gp) or ABCB1, a 170-kDa membrane phosphoglycoprotein encoded by the *mdr*1 gene (*MDR1*) (Germann, 1996). Previous studies indicated that CD147 expression is upregulated in MDR cancer cells, and also demonstrated that CD147 increases the activity of MMPs in MDR-expressing breast cancer cell lines (Yang *et al*, 2003). Treatment of MDR-expressing breast cancers with P-gp substrates can adversely affect therapeutic outcomes through modulation of CD147, MMP-2, MMP-9 and EGFR production (Li *et al*, 2007). Hyaluronan (HA) production in mammary carcinoma cells is also increased by CD147, with MDR being induced in an HA-dependent manner (Marieb *et al*, 2004). Expression of CD44 and MDR1/P-gp seems to be co-regulated, as modulation of CD44 expression correspondingly affects MDR1/P-gp expression in breast cancer (Miletti-González *et al.*, 2005). Previous studies indicate that both CD44 and HA are involved in chemotherapeutic drug resistance in many cancer types (Misra *et al*, 2005; Ohashi *et al*, 2007), but regulation of MDR by CD147 and CD44 in CaP remains to be fully defined.

Tumour cell invasion and development of MDR are associated with hypoxia and low tumour pH. Several studies show a direct relationship between increased cancer cell glucose uptake, glycolysis and tumour aggressiveness. Non-invasive spectroscopy imaging for hyperpolarised lactate also shows elevated lactate for high-grade CaP in a transgenic mouse model, compared with normal prostate (Albers *et al*, 2008). Tumour cell expression of MCT1 and MCT4 has been reported to be regulated by CD147 (Kirk *et al*, 2000). Specifically, interaction of CD147 with MCT1 or MCT4 within the endoplasmic reticulum is necessary for MCT trafficking to the plasma membrane; without CD147, MCTs are degraded and thus non-functional (Gallagher *et al*, 2007). However, the relationship of MCTs with CD147, CD44 and MDR1 in CaP is still unclear.

In this study, we investigated whether there is an association between markers of metastatic potential (CD147, CD44v3-10), MDR-related protein (MDR1) and monocarboxylate transporters (MCT1 and MCT4), and CaP progression and chemoresistance. We found colocalisation of CD147, CD44v3-10, MDR1, MCT1 and MCT4 in metastatic CaP cells lines; Expression of CD147 and CD44v3-10 in metastatic CaP cells was inversely related to docetaxel sensitivity (IC_50_). In addition, we confirmed the colocalisation of CD147 and CD44v3-10 with MDR1, MCT1 and MCT4 in low- and high-grade primary CaP tissues, and demonstrated that overexpression of CD44v3-10, MDR1 and MCT4 is related to clincopathological markers of CaP progression. Our results suggest that CD147 and CD44v3-10 are associated with CaP drug resistance and metastasis, and could be useful therapeutic targets to prevent the development of incurable, recurrent and drug-resistant CaP.

## Materials and methods

### Antibodies

The following antibodies and conjugates were used: mouse anti-EMMPRIN/CD147 monoclonal antibody (MAb) (8D6), rabbit polyclonal anti-human MDR1 antibody (sc-1517-R), rabbit polycolonal anti-MCT1 antibody (H-70), rabbit polycolonal anti-MCT4 (H-90) (Santa Cruz Biotechnology Inc, Santa Cruz, CA, USA); goat polyclonal anti-CD44v3-10 antibody (Alexis Biochemicals, San Diego, CA, USA); Alexa Fluor-488 goat anti-mouse IgG, Alexa Fluor-488 donkey anti-goat IgG, AlexaFluor-594 goat anti-rabbit IgG, Alexa Fluor-594 donkey anti-goat IgG (Molecular Probes, Eugene, OR, USA); biotinylated swine anti-goat, mouse, rabbit immunoglobulins (Igs), streptavidin/HRP, mouse anti-human IgG_1_ negative control antibodies (Dako, Glostrup, Denmark); mouse anti-human MDR1 MAb (F4) mouse anti-*β*-tubulin MAb and goat or rabbit Ig (Sigma-Aldrich Pty Ltd, Castle Hills, NSW, Australia).

### Cell lines and cell culture

Androgen-non-responsive (PC-3-RX-DT2R, PC-3, DU145) and androgen-responsive (LNCaP-LN3, DuCaP) CaP cell lines from different sources were studied (Supplementary Table 1s). Tissue culture reagents were supplemented with 10% (v/v) heat-inactivated fetal bovine serum (Invitrogen Australia Pty Ltd, Melbourne, VIC, Australia), unless otherwise stated. PC-3-RX-DT2R, PC-3 and DU145 cells were cultured in RPMI-1640; LNCaP-LN3 cells in 1 : 1 RPMI-1640:F12-K; and DuCaP cells in DMEM. All were grown in a humidified incubator at 37 °C with 5% CO_2_. After 48 h culture, sub-confluent cells were rinsed twice with Dulbecco's phosphate-buffered saline (PBS) (pH 7.2), detached with 0.25% trypsin/0.05% EDTA in PBS at 37 °C, collected by centrifugation and resuspended in buffer (see below).

The PC-3-RX-DT2R cell line was developed by Russell's group, by exposing xenografts of PC-3 to three doses of docetaxel at 12.5 mg kg^–1^ at 5-day intervals, intravenously, allowing the tumours to regress and then retreating the mice after their regrowth. Tumours that regrew after the second round of treatment were used to establish a line in culture. The cells were cultured *in vitro* with continuous exposure for 7 days to docetaxel at 1–1.25 × 10^−9^ M, followed by a 14-day recovery period in the absence of added docetaxel through three rounds of treatment to establish them as a drug-resistant cell line, PC-3-RX-DT2R. DuCaP cells were provided by Dr K Pienta (University of Michigan Comprehensive Cancer Center, Ann Arbor, MI, USA). PC-3 and DU145 CaP cell lines were obtained from American Type Culture Collection (ATCC, Rockville, MD, USA). LNCaP-LN3 cells were kindly provided by Dr C Pettaway (M. D. Anderson Hospital, Austin, TX, USA).

### Immunofluorescence confocal microscopy analysis of CaP cell lines

To determine the cellular localisation of CD147, CD44v3-10, MDR1, MCT1 and MCT4 in CaP cells, PC-3-RX-DT2R, PC-3, DU145, LNCaP-LN3 and DuCaP cells were grown on glass coverslips (10^5^ cells) for 24 h. After washing with Tris-buffered saline (TBS) (pH 7.5), cells were fixed on coverslips in ice-cold methanol for 10 min at room temperature (RT) and then incubated with 10% normal goat serum in TBS for 20 min to suppress non-specific binding of IgG. After rinsing in TBS, the cells were incubated in mouse anti-CD147 (1 : 400 dilution), goat anti-CD44v3-10 (1 : 400 dilution), rabbit anti-MDR1 (1 : 400 dilution), MCT1 (1 : 400 dilution) and rabbit anti-MCT4 (1 : 400 dilution) antibodies for 1 h at RT on a shaking table and rinsed with TBS, followed by a 45-min incubation in Alexa Fluor-488 goat anti-mouse, donkey anti-goat or Alexa Fluor-594 goat anti-rabbit IgG (1 : 1000 dilution) at RT. The stained cells were mounted with glass slides using glycerol (Sigma-Aldrich Pty Ltd.). Slides were examined using an FV300/FV500 Olympus laser scanning confocal microscope (Olympus, Tokyo, Japan). Negative control slides were treated identically but with either isotype control MAbs or by omitting primary antibodies. We used a constant setting for laser power and detector gain for confocal microscopy. Multichannel excitation was minimised using fluorochromes with peak excitation of 488 and 594 nm, respectively. Emission bleed-through was minimised using multitrack methods in which sequential image capture with a single detection channel was performed and images then combined. This corrects for the effects of emission crosstalk.

### Western blot analysis

Protein expression levels were determined by western blot analysis. Briefly, cells were lysed in a buffer containing 50 mmol l^–1^ Tris–HCl (pH 8.0), 150 mmol l^–1^ sodium chloride (NaCl), 0.1% SDS, 10 mmol l^–1^ sodium fluoride (NaF), 1 mmol l^–1^ sodium orthovanadate (Na3VO4), 0.5% sodium deoxycholate, 1% Triton X-100 and 1/12 (v/v) protease inhibitor cocktail. The lysates were centrifuged at 13000 r.p.m. for 10 min at 4 °C and the supernatants were collected for determining protein concentration using a BCA protein assay reagent (Thermo Scientific, Waltham, MA, USA). Equal amounts of total protein were separated by NuPAGE 4–12% Bis–Tris gel (Invitrogen Australia Pty Ltd) electrophoresis at 200 V for 50 min and then transferred to a PVDF membrane in NuPAGE transfer buffer at 30 V for 1.5 h. Membranes were blocked with either 5% bovine serum albumin (Sigma-Aldrich Pty Ltd) or 5% skim milk in PBS/0.05% Tween 20 buffer. Blots were incubated overnight with specific antibodies at appropriate concentrations (CD147 1 : 300, CD44v3-10 1 : 100, mouse anti-MDR1 (F4) 1 : 500, MCT1 1 : 200 and MCT4 1 : 300 dilution) at 4 °C. After washing for 3 × 10 min in PBS/0.05% Tween 20 buffer, blots were then incubated for another 1 h with an HRP-conjugated IgG secondary antibody (Santa Cruz Biotechnology Inc, 1 : 5000 dilution). After washing for 3 × 10 min in PBS/0.05% Tween 20 buffer, immunoreactive bands were detected using ECL western blotting substrate (SuperSignal West pico Substrate, Thermo Scientific), followed by exposure to film and photographic development. To confirm equal loading of protein lysates, membranes were stripped (Restore Western Blot Stripping Buffer, Thermo Scientific) and re-probed using mouse anti-*β*-tubulin MAb (1 : 10000 dilution), then processed as above. Films were scanned and processed in Adobe Photoshop.

### MTT assay

PC-3-RX-DT2R, PC-3, DU 145, LNCaP-LN3 and DuCaP cells were seeded in triplicate in 96-well plates at 5000 cells per well and incubated for 24 h. A range of concentrations of docetaxel (Sigma-Aldrich, St Louis, MO, USA) diluted in 100% ethanol (1000, 100, 10, 1, 0.1, 0.01, 0.001, 0.0001 nM) was added to the cells. Control cells were treated with appropriate volumes of 100% ethanol. After 48 h, 20 *μ*l of MTT (5 mg ml^–1^) (Sigma-Aldrich Pty Ltd.) was added to each well, followed by incubation at 37 °C/5% CO_2_ for 4 h. Subsequently, 100 *μ*L of DMSO (Sigma-Aldrich Pty Ltd.) was added and the plate was shaken for 20 min at RT to dissolve the formazan crystals. The absorbance (OD) was read at a wavelength of 562 nm on a BIO-TEK microplate reader (Bio-Rad, Hercules, CA, USA). Each experiment was repeated at least three times. Results represent the OD ratio of treated and untreated cells. The growth inhibition curve was generated using the GraphPad Prism 4 Program (GraphPad, San Diego, CA, USA). Absolute IC_50_ values were calculated using the intersection of the 50% normalised drug response and the growth inhibition curves for each cell line, to find the *x* axis values for IC_50_ docetaxel concentration (nM) (also log_10_(nM)).

### Patients and clinical data

As described previously (Cozzi *et al*, 2006), 140 CaP tissues were obtained with informed consent from patients with localised CaP undergoing radical resection of the prostate (RRP) or transurethral resection of the prostate at Urology Sydney, St George Private Hospital, from 2000 to 2007. Controls (*n*=40) were from normal biopsy samples or from morphologically normal areas of CaP tissue. Ethical approval was obtained from the South East Area Health Human Research Ethics Committee, South Section. Specimens were grouped as follows: *Group I:* normal prostate glands (age <40 years, range 26–38 years, *n*=10; age >50 years, range=55–83 years, *n*=10), normal areas of prostate glands from CaP patients (median age 67 years, range 62–84 years, *n*=20), benign prostate hyperplasia (BPH) (median age 66 years, range 58–72 years, *n*=40), prostatic intraepithelial neoplasia (PIN) (median age 63 years, range 57–71 years, *n*=20); *Group II:* 120 CaP specimens (96 RRP, 24 transurethral resection of the prostate), containing Gleason score <7 (*n*=30), Gleason score=7 (3+4) (*n*=30), Gleason score=7 (4+3) (*n*=30), Gleason score >7 (*n*=30), with median age 61 years (range 46–76 years).

Formalin-fixed tissues were routinely processed, paraffin-embedded and H&E sections were reviewed. Tumour foci were identified, circled in ink and graded (Gleason system). Pathological stage (RRP) was determined using the TNM system. Clinical data in RRP patients (*n*=96) indicated an average age at surgery of 63 years (range 49–72 years) and median follow-up time of 18 months (range 2–50 months). A detectable level of PSA (>0.2 ng ml^–1^) after surgery was defined as biochemical recurrence (Cozzi *et al*, 2006). Pertinent clinical information (pretreatment PSA level, Gleason score, clinical stage, surgical margin status, assessment by clinic visit, phone or e-mail contact to determine overall, cancer-specific and recurrence-free survival) was recorded. All patients were advised to undergo a serum PSA test twice a year.

### TMAs

Tissue microarrays (TMAs) were constructed (Cozzi *et al*, 2006) with three tissue cores (diameter 1.0 mm)/donor block within the marked areas, being arrayed into a recipient paraffin block (35 mm × 20 mm) of semiautomated Beecher Instruments (Silver Springs, MD, USA). Sections (5 *μ*m) were cut, collected on Superfrost Plus slides (Lomb Scientific, Sydney, NSW, Australia) and H&E staining was performed.

### Immunohistochemistry

To examine for expression of CD44v3-10, MDR1, MCT1 and MCT4 immunoreactivity, paraffin-embedded TMAs were deparaffinised in xylene, followed by a graded series of ethanol (100, 95, 75 and 50%) and re-hydration in TBS. Slides were immersed in 0.01 M citrate buffer (pH 6.0) for 20 min at 100 °C to enhance antigen retrieval, rinsed in TBS and then treated with 3% hydrogen peroxide, and again rinsed in TBS. After blocking in 10% normal swine serum in TBS for 30 min, sections were incubated overnight at 4 °C in goat anti-CD44v3-10 (1 : 200 dilution), rabbit anti-MDR1 (1 : 200 dilution), rabbit anti-MCT1 (1 : 200 dilution) and rabbit anti-MCT4 (1 : 200 dilution) Polyclonal antibodies (PAbs), washed in TBS, then incubated in biotinylated swine anti-goat or rabbit Ig (1 : 150) for 45 min at RT, rinsed in TBS and then incubated in streptavidin/HRP (1 : 200) for 30 min at RT. After rinsing in TBS, immunoreactivity was developed with 3,3′ diaminobenzidine substrate and counterstained with haematoxylin. Negative controls were treated identically but incubated in control PAbs (nonspecific goat or rabbit Ig), or the primary antibody was omitted.

### Immunofluorescence staining of CaP tissues

To examine the colocalisation of CD147, CD44v3-10, MDR, MCT1 and MCT4 in CaP tissues, whole sections (10 CaP specimens from each subgroup based on TMA immunohistochemistry results, *n*=40) were incubated overnight at 4 °C in primary mouse anti-CD147 (1 : 200 dilution) MAb, or in goat anti-CD44v3-10 (1 : 200 dilution), rabbit anti-MDR1 (1 : 300 dilution), rabbit anti-MCT1 (1 : 200 dilution) and rabbit anti-MCT4 (1 : 200 dilution) PAbs. After washing with TBS, sections were incubated in goat anti-mouse Alexa 488 (CD147), goat anti-rabbit Alexa 594 (MDR1, MCT1 and MCT4) and donkey anti-goat Alexa 488 or 594 (for goat CD44v3-10) for 1 h at RT, and rinsed in TBS. Controls were treated identically, using nonspecific Igs (goat or rabbit Ig) as negative controls. Sections were examined using an FV 300/FV500 Olympus laser scanning confocal microscope (Olympus). We used a constant setting for laser power and detector gain for confocal microscopy, and multitracking and sequential image capture was used to correct signal emission crosstalk between neighbouring channels, and the images were combined.

### Assessment of immunostaining results

Immunostaining results were assessed by staining intensity (Grade 0–3) for cancer cell lines, TMA tissue and whole primary prostate and CaP tissues using light microscopy (Leica microscope, Nussloch, Germany) and confocal microscopy. The criteria for assessment were as follows: 0, negative); 1, weak); 2, moderate); 3, strong). For TMA staining, three cores were scored per case. The analysis of three cores per case has been shown to be comparable with the analysis of the whole section in a previous study (Rubin *et al*, 2002). In instances in which all three cores from one tumour were positive (3 of 3), the reading was counted as positive. In situations in which heterogeneous staining was seen among the three cores, an average score was determined. Evaluation of tissue staining was performed independently by three experienced observers (JLH, HMC and YL). All specimens were scored blind and an average of grades was taken. If discordant results were obtained, differences were resolved by joint review and consultation with a third observer (WD) experienced in immunopathology. For statistical analysis, CaP patients from RRP cases were divided into two groups: the low-expression group, comprising Grade 0 and 1 immunostaining, and the high-expression (overexpression) group, comprising Grade 2 and 3 immunostaining.

### Statistical analysis

The associations between CD44v3-10, MDR1 and MCT4 expression levels (low-expression and high-expression groups) and clinicopathological data were tested using a *χ*^2^-test. Comparison of staining intensity for CD44v3-10, MDR1, MCT1 and MCT4 between CaP tissues and normal prostate tissues was performed using the *χ*^2^-test, where *P*<0.05 (two tailed) was considered significant. All statistical analyses were performed using GraphPad Prism 4.00 (GraphPad).

## Results

### Expression and colocalisation of CD147, CD44v3-10, MDR1, MCT1 and MCT4 in metastatic and drug-resistance CaP cell lines

**Figure 1:**
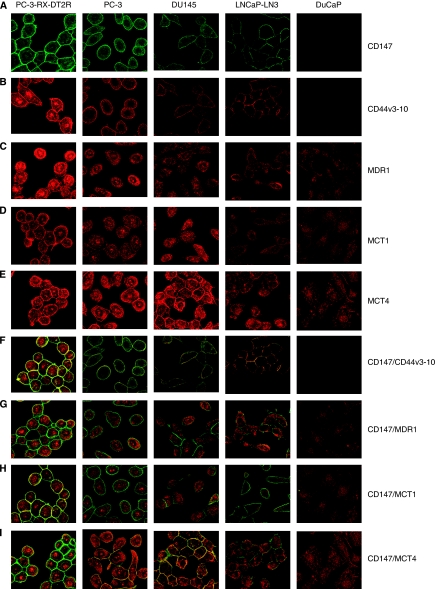
Co-immunolabelling of CD147, CD44v3-10, MDR1, MCT1 and MCT4 in metastatic prostate cancer (CaP) cell lines. Representative confocal images of CD147 (green), CD44v3-10, MDR1, MCT1 and MCT4 (red) expression are shown. Merged images and red and green channels are shown separately. Membrane expression was found for CD147 and CD44v3-10 antibodies, whereas expressions of both membrane and cytoplasm were seen for MDR1, MCT1 and MCT4 antibodies. All immunostainings are more homogeneous. (**A**) CD147; (**B**) CD44v3-10; (**C**) MDR1; (**D**) MCT1; (**E**) MCT4; (**F**) colocalisation of CD147 with CD44v3-10; (**G**) colocalisation of CD147 with MDR1; (**H**) colocalisation of CD147 with MCT1; (**I**) colocalisation of CD147 with MCT1. Magnification: **A**–**I** × 400. The colour reproduction of the figure is available on the html full text version of the paper.

**Figure 2:**
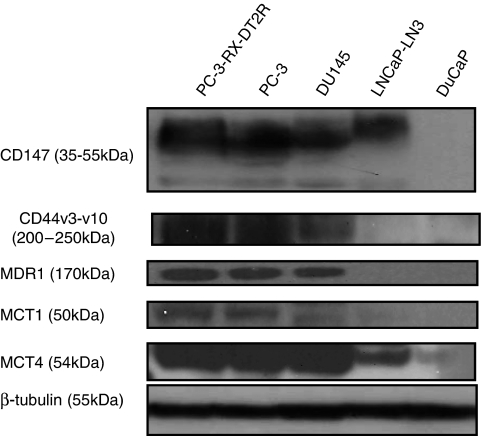
Expression of CD147, CD44v3-10, MDR1, MCT1 and MCT4 in prostate cancer (CaP) cell lines by western blotting. A representative western blot showing high levels of CD147, CD44v3-10, MDR1 (F4), MCT1 and MCT4 expression in PC-3-RX-DT2R and PC-3 cell lines, moderate expression in DU 145 cells and low or no expression in LNCaP and DuCaP cells. Equal loading is demonstrated with *β*-tubulin antibody in the bottom panel (lane 1: RX-DT2R, lane 2: PC-3, lane 3: DU145, lane 4: LNCaP-LN3. lane 5: DuCaP).

Immunofluorescence labelling of CaP cells with CD147, CD44v3-10, MDR1, MCT1 and MCT4 antibodies showed positive staining in PC-3-RX-DT2R, PC-3, DU145 and LNCaP-LN3 CaP cells, with variation between cell lines (Figure 1). Strong (Grade 3) expression of CD147, CD44v3-10, MDR1, MCT1 and MCT4 expression was found in PC-3-RX-DT2R and PC-3 cell lines. Medium expression of CD147, CD44v3-10, MDR1 and MCT1 and strong expression of MCT4 were found in DU145 cells. Low expression of CD147, CD44v3-10, MDR1 and MCT1 and medium expression of MCT4 were found in the LNCaP-LN3 cell line. DuCaP cells showed no staining for CD147 and CD44v3-10, and weak immunostaining for MDR1, MCT1 and MCT4. The immunostaining grades are summarised in Supplementary Table 2s. Membrane expression was found for CD44v3-10 PAb, whereas expression of both membrane and cytoplasm was seen for CD147, MDR1, MCT1 and MCT4 antibodies. Strong colocalisation of CD147/CD44v3-10, CD147/MDR1, CD147/MCT1, CD147/MCT4, CD44v3-10/MDR1, CD44v3-10/MCT1 and CD44v3-10/MCT4 was observed in PC-3-RX-DT2R, PC-3 and DU 145 non-androgen-responsive metastatic CaP cell lines; weak colocalisation of these markers was observed in the LNCaP-LN3 cell line but no colocalisation was found in the DuCaP androgen-responsive CaP cell line (Figure 1 and Supplementary Figure 1s). The immunofluorescence results for the expression of CD147, CD44v3-10, MDR1, MCT1 and MCT4 in CaP cell lines were further confirmed by western blotting and high levels of these proteins were found in PC-3-RX-DT2R and PC-3 cell lines (Figure 2).

### Expression of CD147 and CD44v-3-10 is related with docetaxel response in metastatic CaP cell lines

**Figure 3:**
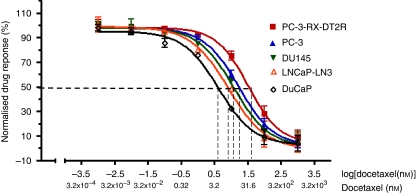
Dose response of metastatic prostate cancer (CaP) cell lines to docetaxel measured using 3-(4,5-dimethylthiazol-2-yl)-2,5-diphenyl tetrazolium bromide (MTT) assay. Drug-resistant and metastatic CaP cell lines treated with a range of concentrations of docetaxel (0.001–1000 nM, *x* axis) showed varying responses. The IC_50_ value (50% normalised cell response) is related to the expression of CD147 and CD44v3-10. For example, the drug-resistant cell line, PC3-RX-DT2R, displays Grade 3 CD147 and CD44v3-10 expression, and an IC_50_ of 44.7 nM (*x* axis); the drug-sensitive CaP cell line, DuCaP, displays Grade 0 CD147 and CD44v3-10 immunostaining and the lowest IC_50_ of 4 nM (*x* axis) (*n*=3, mean±s.d.).

Metastatic CaP cell lines (PC-3-RX-DT2R, PC-3, DU145, LNCaP-LN3 and DuCaP) with different levels of CD147 and CD44v3-10 expression responded differently to docetaxel treatment. The IC_50_ values for these CaP cell lines were highly related to the levels of CD147 and CD44v3-10 expression (see Figure 3 and Supplementary Table 2s). Thus, PC-3-RX-DT2R drug-resistant cells (CD147 and CD44v3-10, Grade3) were the least sensitive (IC_50_: 44.7 nM), whereas DuCaP cells (CD147 and CD44v3-10, Grade 0) were very sensitive to docetaxel treatment (IC_50_: 4 nM).

### Expression of CD44v3-10, MDR1, MCT1 and MCT4 in CaP tissues

**Figure 4:**
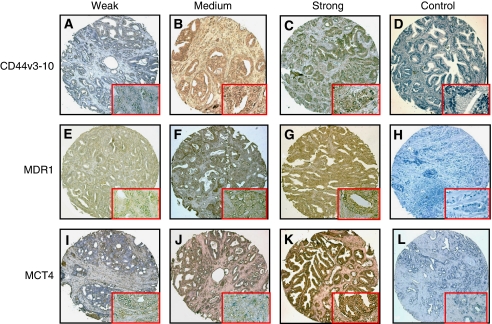
Expression of CD44v3-10, MDR1 and MCT4 in prostate cancer (CaP) tissue microarrays (TMAs). Representative images are shown of Grade 1 (weak) CD44v3-10 (**A**), MDR1 (**E**) and MCT4 (**I**); Grade 2 (medium) CD44v3-10 (**B**), MDR1(**F**) and MCT4 (**J**); and Grade 3 (strong) CD44v3-10 (**C**), MDR1(**G**) and MCT4 (**K**) immunostaining. No immunoreactivity is seen in non-specific negative controls for CD44v3-10 (**D**), MDR1 (**H**) and MCT4 (**L**). Brown colour indicates positive immunostaining. Insets indicate the typical areas of staining at high amplification. Magnification: **A**–**L** × 10; and insets × 40. The colour reproduction of the figure is available on the html full text version of the paper.

We previously reported CD147 expression in these TMAs (Madigan *et al*, 2008). In this study, we immunostained for CD44v3-10, MDR1, MCT1 and MCT4. In primary CaP tissues, 74 (89 of 120), 78 (94 of 120), 88 (106 of 120) and 92% (110 of 120) were positive for CD44v3-10, MDR1, MCT1 and MCT4 (Grade 1–3), respectively. In CD44v3-10-positive primary CaP sections, weak staining (Grade 1) was found in 16% (14 of 89) (Figure 4A), moderate staining (Grade 2) in 45% (40 of 89) (Figure 4B) and strong staining (Grade 3) in 39% (35 of 89) (Figure 4C), whereas no staining was observed in negative controls (Figure 4D).

In MDR1-positive primary CaP sections, weak staining (Grade 1) was found in 17% (16 of 94) (Figure 4E), moderate staining (Grade 2) in 44% (41 of 94) (Figure 4F) and strong staining (Grade 3) in 39% (37 of 94) (Figure 4G), whereas no staining was found in negative controls (Figure 4H).

The immunostaining patterns and percentage of positive cells for MCT1 and MCT4 in TMAs were similar, and MCT4 results are presented as representative of this study. In MCT4-positive primary CaP sections, weak staining (Grade 1) was found in 20% (22 of 110) (Figure 4I), moderate staining (Grade 2) in 38% (42 of 110) (Figure 4J) and strong staining (Grade 3) in 42% (46 of 110) (Figure 4K), whereas no staining was found in negative controls (Figure 4L).

No CD44v-3-10, MDR1, MCT1 and MCT4 immunostaining was found in normal prostate tissues and PIN and in non-tumour regions from primary CaP tissues (data not shown). Scattered areas of weak (⩽Grade 1) heterogeneous epithelial cell staining were observed in 3% (1 of 40) for MDR1, and in 5% (2 of 40) for MCT4 in BPH specimens (Supplementary Table 3s). The staining intensity and percentage of positive staining for CD44v3-10, MDR1, MCT1 and MCT4 in primary CaP tissues, PIN, BPH and normal prostates are summarised in Supplementary Table 3s and Supplementary Table 4s. For CaP specimens, the immunostaining was mostly Grade 2 or 3, but was negative in PIN specimens.

Expression of CD44v3-10, MDR1, MCT1 and MCT4 was generally uniform in most tumours. The expression of CD44v3-10 was mostly cell membrane associated; however, distinct positive cytoplasmic staining was also seen. Immunostaining for MDR1, MCT1 and MCT4, as well as some membrane staining, was mainly cytoplasmic. In high-grade primary CaP (Gleason score ⩾7), the tumour stroma generally showed a strong positive reaction for CD44v3-10, MDR1, MCT1 and MCT4 (data not shown).

### Correlation between CD44v3-7, MDR1 and MCT4 expression and clinicopathological parameters

Of the 96 RRP patients, only 9% (9 of 96) relapsed with biochemical progression (PSA>0.4), and no patients died of CaP during the follow-up period (5 years). The median time to relapse was 40 months (range 18–50 months). In all, 26% (25 of 96) of tumours had a Gleason score <7 whereas 74% (71 of 96) of tumours had a Gleason score ⩾7. In all, 27% (26 of 96) of tumours were small (pT1), 38% (36 of 96) were organ confined (stage pT2) and 36% (34 of 96) had extracapsular extension (stage pT3). Table 1 summarises the correlations between CD44v3-10, MDR1 and MCT4 expression in primary CaP with a pretreatment PSA level, Gleason score, pathological stage, surgical margin status, nodal involvement (development of metastases) and biochemical recurrence. Overexpression (high-expression group) of CD44v3-10, MDR1 and MCT4 was significantly correlated with pretreatment PSA levels (*P*<0.05), and increased with progression of CaP (Gleason score, *P*<0.05; pathological stage, *P*<0.05; nodal involvement, *P*<0.05). Overexpression of CD44v3-10 and MDR1, but not of MCT4, was also significantly correlated with PSA-defined recurrence (*P*<0.05). There was no correlation between overexpression of CD44v3-10, MDR1 or MCT4 and surgical margin (*P*>0.05).

### Co-immunolabelling of primary CaP tissues with CD147, CD44, MDR1, MCT1 and MCT4 antibodies

**Figure 5:**
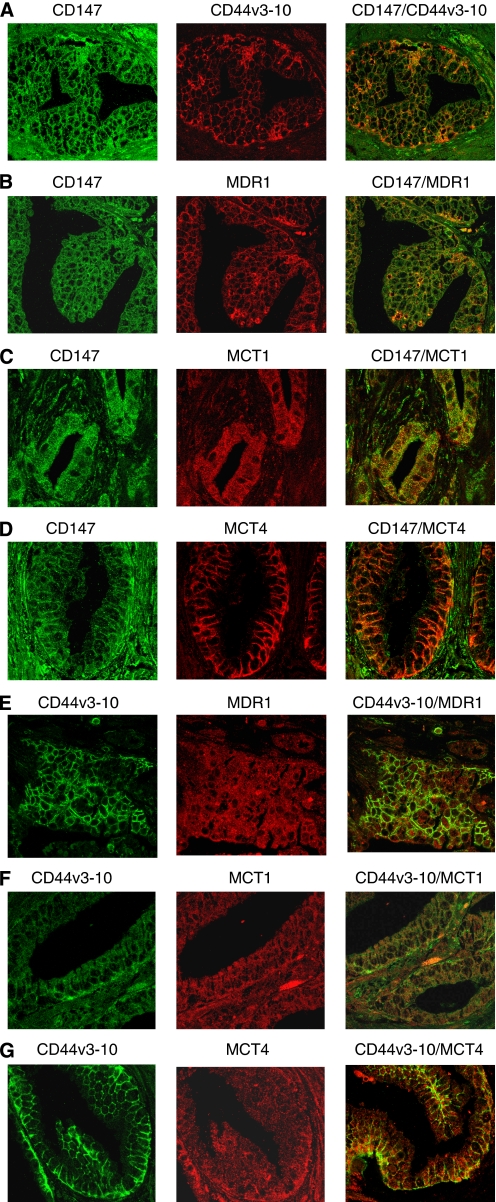
Co-immunolabelling of CD147, CD44v3-10, MDR1, MCT1 and MCT4 in primary prostate cancer (CaP) tissues (different tumours with a range of Gleason scores). Representative confocal images of CD147 and CD44v3-10 (green), CD44v3-10, MDR1, MCT1 and MCT4 (red) immunolabelling are shown. Merged images and red and green channels are shown separately. (**A**) CD147 and CD44v3-10 in high-grade CaP (Gleason score=8); (**B**) CD147 and MDR1 in high-grade CaP (Gleason score=8); (**C**) CD147 and MCT1 in low-grade CaP (Gleason score=6); (**D**) CD147 and MCT4 in high-grade CaP (Gleason score=8); (**E**) CD44v3-10 and MDR1 in high-grade CaP (Gleason score=9); (**F**) CD44v3-10 and MCT1 in high-grade CaP (Gleason score=8); (**G**) CD44v3-10 and MCT4 in high-grade CaP (Gleason score=8). CD147 immunolabelling is seen on epithelial cell membranes and stromal cells. CD44v3-10, MDR1, MCT1 and MCT4 immunostaining is predominantly epithelial. MCT4 localises mostly to basal epithelial cells and to lateral cell walls (magnification: **A**–**G** × 400). The colour reproduction of the figure is available on the html full text version of the paper.

Colocalisation of CD147 and CD44v3-10, MDR1, MCT1and MCT4 was also assessed in primary CaP tissues (*n*=40) by confocal microscopy. Most samples displayed co-immunolabelling with two different markers, although immunostaining for single antibody in different samples was variable. The immunostaining patterns are very similar to those seen by peroxidase immunohistochemistry as described above. Representative images from different tumours are shown in Figure 5. For co-immunolabelling of CD147 with CD44v3-10 (Figure 5A), CD147 with MDR1 (Figure 5B), CD147 with MCT1 (Figure 5C), CD147 with MCT4 (Figure 5D), CD147 is green, whereas CD44v3-10, MDR1, MCT1 and MCT4 expression is red. For co-immunolabelling of CD44v3-10 with MDR1 (Figure 5E), MCT1 (Figure 5F) and MCT4 (Figure 5G), CD44v3-10 is green, whereas MDR1, MCT1 and MCT4 expression is red.

## Discussion

In this study, we examined the expression of CD147, CD44v3-10, MDR1, MCT1 and MCT4 in metastatic CaP cell lines, in primary CaP, PIN, BPH and normal prostate tissues using a tissue bank, and investigated further possible associations among these markers. High levels of CD147, CD44v3-10, MDR1, MCT1 and MCT4 were observed in metastatic and drug-resistant CaP cell lines and in specimens of advanced CaP, but not in PIN, BPH and normal prostate tissues. Colocalisation of invasive and metastatic markers (CD147, CD44v3-10), MDR-related protein (MDR1) and monocarboxylate transporters (MCT1 and MCT4) was also found in most metastatic CaP cell lines, as well as in primary CaP tissues. To our knowledge, this is the first report investigating the relationship between CD147, CD44v3-10, MDR1, MCT1 and MCT4 during CaP progression.

The colocalisation of CD147 separately with several different molecules, such as CD44v3-10, MDR1, MCT1 and MCT4, and the colocalisation of CD44v3-10 with MDR1, MCT1 and MCT4 in primary and metastatic CaP cells, suggests interactions between these proteins. Toole and Slomiany (2008) reported that CD147 and CD44 interact with various multidrug transporters of the ABC family and with MCTs associated with resistance to cancer therapies. Slomiany *et al* (2009) reported that CD44 colocalises with MCT1, MCT4 and CD147 at the plasma membrane, and HA, CD44 and CD147 contributed to the regulation of MCT localisation and function in the plasma membrane of breast cancer cells (Slomiany *et al*, 2009). Colocalisation of CD44 and MDR1 was shown to increase in melanoma cells engineered to express MDR, compared with parental cells (Colone *et al*, 2008). Su *et al* (2009) also found that CD147 colocalises with MCT1 and MCT4 in membranes of malignant A375 melanoma cells, leading to an increased glycolytic rate compared with that in normal human melanocytes. Silencing of CD147 in A375 cells abrogates expression of MCT1 and MCT4, and colocalisation with CD147, and dramatically decreases the cellular glycolytic rate, extracellular pH and the production of ATP (Su *et al*, 2009). Our present data show colocalisation of CD147 with MCT1 and MCT4 in primary and metastatic CaP cells, consistent with CD147 being an ancillary protein required for the expression of these MCTs (Deora *et al*, 2005; Gallagher *et al*, 2007). Our results support the hypothesis that expression of CD147 is closely related to that of CD44v3-10, and may be involved in regulating the expression of MDR1, MCT1 and MCT4 during CaP metastasis.

The expression of CD44 and its variants is associated with the progression of several cancers, although this remains controversial for CaP (De Marzo *et al*, 1998; Noordzij *et al*, 1999). One study reported a complete lack of membranous expression of all CD44 isoforms in 93–98% primary CaP tissues (Kallakury *et al*, 1996), whereas another reported moderate to high levels of CD44 expression in 60% of primary CaP, with ∼14% of metastases expressing low levels of CD44 (Nagabhusha *et al*, 1996). Significant reduction in CD44 expression was also reported in primary CaP foci and metastases by De Marzo *et al* (1998). The relationship between CD44 expression and tumour grade is also uncertain, with a strong correlation between the Gleason grade of CaP and loss of CD44 expression in one study (De Marzo *et al*, 1998), but no correlation in another (Paradis *et al*, 1998). Similar to expression studies, the potential role of CD44 in CaP development and metastases is controversial. Earlier, overexpression experiments have suggested that CD44 may exert a tumour-suppressive function (Gao *et al*, 1998), although other studies have implicated CD44 in CaP cell proliferation, adhesion, migration and invasion *in vitro*, as well as in metastatic dissemination *in vivo* (Paradis *et al*, 1998; Omara-Opyene *et al*, 2004). The variation in CD44 expression seen in different studies may be attributable to the use of different methodologies in the assessment of CD44 expression or to the different stages of CaPs used in the analyses or to the use of different antibodies. Differences in the expressed CD44 isoform also explain some of these controversies. Non-invasive prostate epithelial cells have been shown to express a high-molecular-weight CD44 isoform, CD44v3-10, which may counteract the function of the standard isoform of CD44s by reducing adhesion to and invasion of the endothelium by CaP cells (Harrison *et al*, 2006).

In this study, we found CD44s expression in normal prostate tissues and in a very low percentage of cells in CaP tissues (of different stages, Hao J *et al*, unpublished data); CD44v3-10 was negative in all normal prostate and PIN tissues. However, high levels of expression of CD44v3-10 were correlated with tumour grade, clinical stage, residual tumour and relapse, but not with differences in tumour histological type. These observations support the idea that in the development of CaP, CD44 isoform expression changes progressively from CD44s to high-molecular-weight variant forms such as CD44v3-10, and that CD44s basal cell expression is lost with overexpression of variant forms in CaP cells (Hao *et al*, 2010). The data suggest that CD44v3-10 is a marker of progression of prostate epithelial cells from a benign to a malignant phenotype, and thus may be an important indicator of the stage of CaP, reflecting CaP progression and metastasis.

Aberrant MDR1 expression has been seen in many cancer types, including CaP, and contributes significantly to treatment failure. MDR1 expression was found to be associated with drug resistance in androgen-dependent and androgen-independent human prostate xenografts (Chen *et al*, 1998), whereas downregulation of the *MDR1* gene by hypermethylation has been associated with an increase in cellular proliferation possibly related to disease progression (Van Brussel *et al*, 2001; Enokida *et al*, 2004). *In vitro* studies have also reported a functional interaction between CD44 and MDR1, associated with increased cell migration, *in vitro* invasion and metastasis (Miletti-González *et al.*, 2005). Our analysis of primary CaP tumour samples of different stages/grades before drug therapy has shown high levels of MDR1 expression to be correlated with tumour grade, clinical stage, residual tumour and relapse, suggesting that MDR1 expression may be involved in CaP progression and metastasis. We also found that expression of CD147 and CD44v3-10 is colocalised in metastatic CaP cells and inversely related to docetaxel sensitivity in metastatic CaP cell lines, suggesting that CD147 and CD44v3-10 may be involved in CaP drug resistance. The functional roles of CD147 and CD44v3-10 in CaP metastasis and drug resistance are currently being investigated in our laboratory.

Increased glycolysis and adaptation to acidosis are key events in the transition from *in situ* to invasive cancer (Gatenby and Gillies, 2004). Given their essential function in exporting lactate, the end product of glycolysis, MCTs are considered key elements in regulating tumour intracellular pH and in the induction of extracellular acidosis (Pinheiro *et al.*, 2009). The rapid transport of lactate through MCTs is of critical importance for tumour cells, by which an increased glycolytic rate gives a proliferative advantage over other cells. Upregulation of MCTs has been described in several tumour types, but only three studies have evaluated its clinicopathological significance (Pinheiro *et al*, 2008a, 2008b, 2009). In this paper, we demonstrate for the first time that the high level of expression of MCT4 is correlated with CaP tumour grade, clinical stage and residual tumour, as well as with relapse, but not with differences in histological type, consistent with MCT1/MCT4 expression being involved in CaP progression.

We previously demonstrated CD147 expression in metastatic CaP cell lines, primary CaP tissues and lymph node metastases (Madigan *et al*, 2008). This has been ratified in this study, together with overexpression of CD44v3-10, MDR1, MCT1, MCT4 and colocalisation of CD147 and CD44v3-10, with MDR1, MCT1 and MCT4 in CaP and stromal cells (data not shown) in most primary tumours. The colocalisation of these markers in CaP tissues is consistent with that seen in CaP cell lines, suggesting that cancer clones that escape from primary tumours to the common metastatic sites in human CaP do not lose expression of these antigens. Differential expression of CD147, CD44v3-10, MDR1, MCT1 and MCT4 also suggests that the phenotypes of CaP metastasis may be controlled by genetics and/or by the tumour microenvironment during CaP progression. Functional interactions between CD44 and MDR1 are increasingly being recognised as important in tumour metastases. For example, in breast and ovarian cancer cell lines, immunoprecipitation and colocalisation studies, together with functional assays, showed that CD44 and MDR can directly influence the expression of each other, producing a malignant tumour cell phenotype characterised by MDR, increased migration and invasion (Miletti-González *et al.*, 2005). The colocalisation of CD147 with CD44v3-10, MDR1, MCT1 and MCT4 in this study further suggests that CD147 and CD44v3-10 could concomitantly regulate MDR1, MCT1 and MCT4 expression during CaP progression, associated with drug resistance. However, the mechanisms involved in CD147 and CD44v3-10 regulation during CaP metastasis require further study. Given that CD147 and CD44v3-10 colocalise with MDR1-positive cells in CaP specimens, their targeting could potentially overcome drug resistance in the late stage of metastatic CaP.

Previous studies have shown that CD147 knockdown using siRNA (Wang *et al*, 2006) or antibodies (Xu *et al*, 2007; Dean *et al*, 2009) inhibits tumour growth *in vitro* or *in vivo*, associated with changes in the regulation of MMP production (Xu *et al*, 2007, Dean *et al*, 2009) and radiation sensitivity of the tumours (Dean *et al*, 2009). Schneiderhan *et al* (2009) further confirmed that CD147 silencing inhibits lactate transport and reduces malignant potential of pancreatic cancer cells in *in vitro* and *in vivo* models. MCT1 inhibition has also been shown to have anti-tumour potential against *in vivo* models of lung carcinoma, colorectal carcinoma and a squamous carcinoma cell line after *α*-cyano-4-hydroxycinnamate-mediated MCT1 inhibition (Sonveaux *et al*, 2008). These results suggest that targeting CD147 could be useful in controlling metastasis and cancer recurrence, with potential application to CaP.

Targeting overexpressed CD44 in cancer cells may also control CaP progression. Antibody-mediated CD44 targeting has inhibited growth of breast cancer xenografts and prevented regrowth of basal-like HBCx cells after chemotherapy-induced remission (Marangoni *et al*, 2009). Gene therapy using siRNA CD44 also caused *in vitro* and *in vivo* regression of HT colon cancer cells (Subramaniam *et al*, 2007). Several reviews have also discussed the advantages of HA (major CD44 ligand) as a drug carrier and a targeting ligand for cancer, as well as other pathologies (Platt and Szoka, 2008; Yadav *et al*, 2008). It has also been used with prodrugs against cancer cell lines and xenografts (Auzenne *et al*, 2007) or in novel lipoplexes to target siRNA (Taetz *et al*, 2009), or for gene delivery (Surace *et al*, 2009). The potential for these approaches in CaP, alone or in combination with CD147-targeted therapies (discussed above), is promising and remains to be investigated in future studies.

In summary, we have demonstrated co-expression of CD147 and CD44v3-10 with MDR1, MCT1 and MCT4 in most CaP metastatic cell lines and in primary CaP tissues. The overexpression of CD44v3-10, MDR1 and MCT4 was significantly associated with CaP progression. Colocalisation of CD147 and CD44v3-10 with MDR1 and MCTs in tumour and stromal cells suggests a role for these invasive markers in the regulation of drug resistance in the progression of CaP, consistent with our *in vitro* docetaxel sensitivity findings. Our results further indicate that both CD147 and CD44v3-10 may be potential therapeutic targets for treating late-stage, incurable, recurrent metastatic CaP to overcome drug resistance.

## Figures and Tables

**Table 1:** Clinicopathological characteristics associated with CD44v3-10, MDR1 and MCT4 expression in primary CaPs (RRP patients, *n*=96)

	**CD44v3-10, MDR1 and MCT4 expression/total number (%)**								
	**CD44v3-10**			**MDR1**			**MCT4**		
**Variable**	**LEG^a^**	**HEG^b^**	***P*-value^c^**	**LEG**	**HEG**	***P*-value^c^**	**LEG**	**HEG**	***P*-value^c^**
*Pretreatment PSA level (ng ml* ^*–1*^ *)*									
<10	53% (21/40)	47% (19/40)	**0.001**	45% (18/40)	55% (22/40)	**0.014**	35% (14/40)	65% (26/40)	**0.004**
⩾10	21% (12/56)	79% (44/56)		21% (12/56)	79% (44/56)		11% (6/56)	89% (50/56)	
									
*Gleason score*									
<7	52% (13/25)	48% (12/25)	**0.031**	56% (14/25)	44% (11/25)	**0.002**	36% (9/25)	64% (16/25)	**0.029**
⩾7	28% (20/71)	72% (51/71)		23% (16/71)	77% (55/71)		15% (11/71)	85% (60/71)	
									
*Pathological stage*									
pT1	54% (14/26)	46% (12/26)	**0.041**	58% (15/26)	42% (11/26)	**0.002**	42% (11/26)	58% (15/26)	**0.006**
pT2	30% (11/36)	70% (25/36)		25% (9/36)	75% (27/36)		11% (4/36)	89% (32/36)	
pT3	24% (8/34)	76% (26/34)		18% (6/34)	82% (28/34)		15% (5/34)	85% (29/34)	
									
*Nodal involvement*									
No	40% (32/81)	60% (49/81)	**0.014**	36% (29/81)	64% (52/81)	**0.024**	25% (20/81)	75% (61/81)	**0.031**
Yes	7% (1/15)	93% (14/15)		7% (1/15)	93% (14/15)		0% (0/15)	100% (15/15)	
									
*Surgical margin*									
Negative	39% (24/61)	61% (37/61)	0.176	38% (23/61)	62% (38/61)	0.075	25% (15/61)	75% (46/61)	0.232
Positive	26% (9/35)	74% (26/35)		20% (7/35)	80% (28/35)		14% (5/35)	86% (30/35)	
									
*PSA-defined recurrence*									
No	37% (33/89)	62% (54/87)	**0.023**	34% (30/87)	66% (57/87)	**0.034**	23% (20/87)	77% (67/87)	0.106
Yes	0% (0/7)	100% (9/9)		0% (0/9)	100% (9/9)		0% (0/9)	100% (9/9)	

Abbreviations: CaP,=prostate cancers; HEG=high expression group; LEG=low expression group; PSA=prostate-specific antigen; RRP=radical resection of the prostate.

a LEG (Grade 0 or 1 immunostaining).

b HEG (⩾ Grade 2 immunostaining).

c *χ*^2^-test; *P*<0.05 significant. The bold indicates the significant difference between groups in each parameter.

## Supplementary Material

Supplementary Figure

Supplementary Table1

Supplementary Table2

Supplementary Table3

Supplementary Table4
