# Supplementary figures and images for: Co-expression of CD147 (EMMPRIN), CD44v3-10, MDR1 and monocarboxylate transporters is associated with prostate cancer drug resistance and progression
Source: Br J Cancer. 2010 Aug 24;103(7):1008–18. doi: 10.1038/sj.bjc.6605839 (PMC2965856; doi:10.1038/sj.bjc.6605839)

## Slide 1
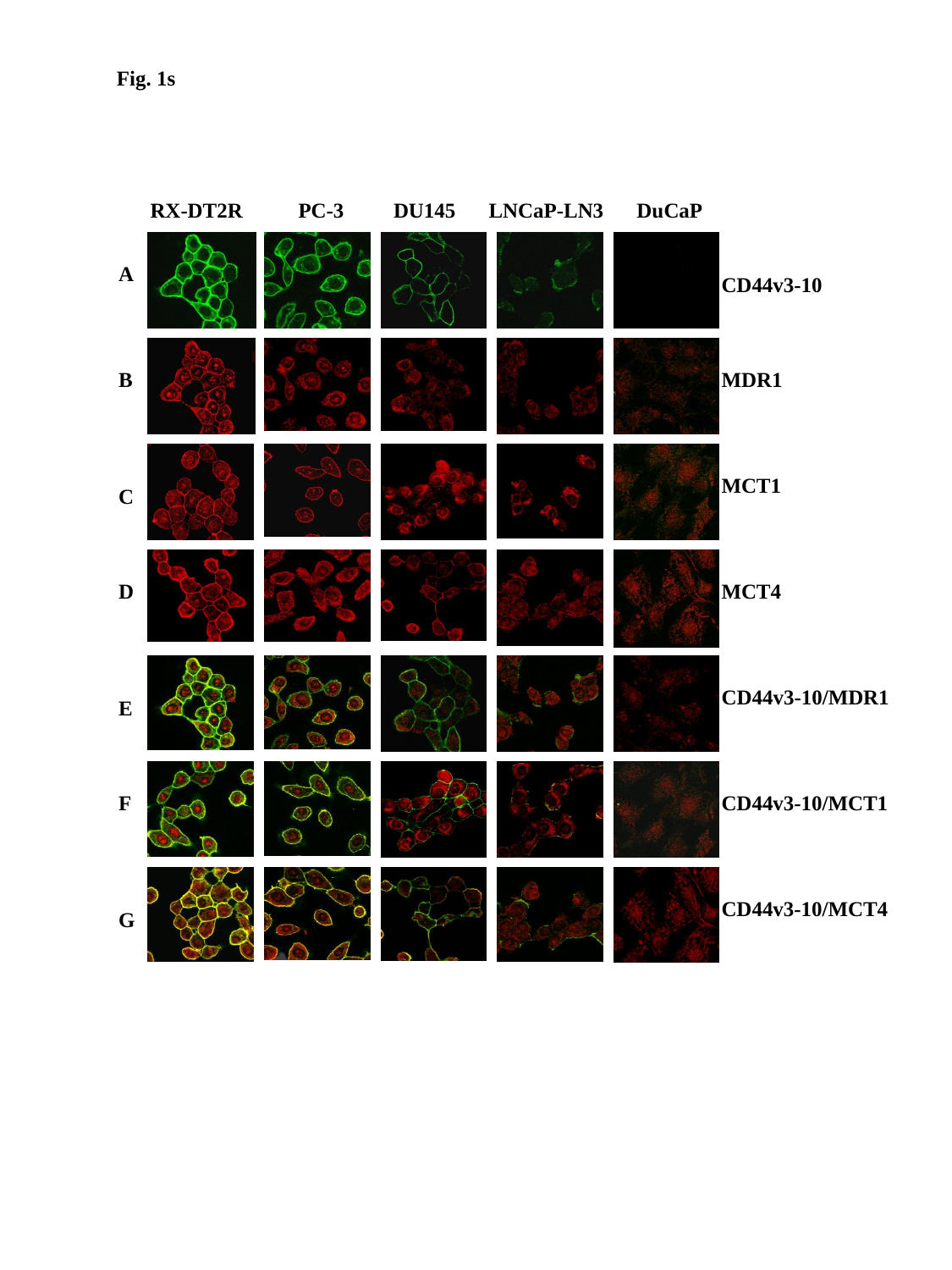

Fig. 1s
RX-DT2R
PC-3
DU145
LNCaP-LN3
DuCaP
A
CD44v3-10
B
MDR1
MCT1
C
D
MCT4
CD44v3-10/MDR1
E
F
CD44v3-10/MCT1
CD44v3-10/MCT4
G

Supplement: Supplementary Figure [file 6605839x1.ppt]
