# Supplementary material for: Co-expression of CD147 (EMMPRIN), CD44v3-10, MDR1 and monocarboxylate transporters is associated with prostate cancer drug resistance and progression
Source: Br J Cancer. 2010 Aug 24;103(7):1008–18. doi: 10.1038/sj.bjc.6605839 (PMC2965856; doi:10.1038/sj.bjc.6605839)
Supplement: Supplementary Table1 [file 6605839x2.doc]

**Table 1s:** Characteristics of CaP Cell Lines

| Cell Line | Site of origin (species) | Source | Androgen  response* |
| --- | --- | --- | --- |
| PC-3-RX-DT2R | PC-3 subline (established from PC-3 xenograft repeatedly exposed to docetaxel in vivo, then exposed to docetaxel in vitro) | Developed by E Kingsley, M Sajinovic, PJ Russell (Oncology Research Centre, Prince of Wales Clinical School, The University of New South Wales | N |
| PC-3 | Bone (human) | ATCC-CRL-1435 | N |
| DU145 | Brain (human) | ATCC-HTB-81 | N |
| LNCaP-LN3 | Lymph node (mouse) | MD Anderson Hospital, Austin Texas, USA | R |
| DuCaP | Dura mater (human) | Hallym University, Seoul, Korea | R |

*****N: nonresponsive to androgen; R: responsive to androgen.
