# Supplementary material for: Co-expression of CD147 (EMMPRIN), CD44v3-10, MDR1 and monocarboxylate transporters is associated with prostate cancer drug resistance and progression
Source: Br J Cancer. 2010 Aug 24;103(7):1008–18. doi: 10.1038/sj.bjc.6605839 (PMC2965856; doi:10.1038/sj.bjc.6605839)
Supplement: Supplementary Table2 [file 6605839x3.doc]

**Table 2s.** Immunostaining for CD147, CD44, MDR1, MCT1 and MCT4 in metastatic CaP cell lines, and response to docetaxel (IC_50_).

|  | **CD147** | **CD44v3-10** | **MDR1** | **MCT1** | **MCT4** | **DCT IC_50_**  **(nM)** |
| --- | --- | --- | --- | --- | --- | --- |
| **Cell Line** |  |  |  |  |  |  |
| PC-3-RX-DT2R | 3 | 3 | 3 | 2 | 3 | 44.7 |
| PC-3 | 2 | 2 | 2 | 2 | 3 | 17.8 |
| DU145 | 1~2 | 1~2 | 2 | 2 | 3 | 10.5 |
| LNCaP-LN3 | 1 | 1 | 1 | 2 | 1.5 | 7.9 |
| DuCaP | 0 | 0 | 0 | 1 | 1 | 4.0 |

**Notes:** **Immunofluorescence staining:** 0= negative; 1= weak; 2= moderate; 3= strong.

**DCT IC_50_:** Docetaxel concentration that reduces cell viability to 50% of control (MTT assay; n=3)
