# Supplementary material for: Co-expression of CD147 (EMMPRIN), CD44v3-10, MDR1 and monocarboxylate transporters is associated with prostate cancer drug resistance and progression
Source: Br J Cancer. 2010 Aug 24;103(7):1008–18. doi: 10.1038/sj.bjc.6605839 (PMC2965856; doi:10.1038/sj.bjc.6605839)
Supplement: Supplementary Table3 [file 6605839x4.doc]

**Table 3s**. Percentage positive immunostaining for CD44v3-10, MDR1, MCT1 and MCT4 in normal prostate, BPH and different grades of CaP (n=120)

|  | **CD44v3-10** | **MDR1** | **MCT1** | **MCT4** |
| --- | --- | --- | --- | --- |
| Group I |  |  |  |  |
| Normal prostate (n=40) | 0 | 0 | 0 | 0 |
| BPH (n=40) | 0 | 3% (1/40) | 0 | 5% (2/40) |
| PIN (n=20) | 0 | 0 | 0 | 0 |
| Group II |  |  |  |  |
| G < 7 (n=30) | 60% (18/30) | 73% (22/30) | 83% (25/30) | 87% (26/30) |
| G = 7 (3+4) (n=30) | 73% (22/30) | 77% (23/30) | 83% (25/30) | 90% (27/30) |
| G = 7 (4+3) (n=30) | 77% (23/30) | 80% (24/30) | 93% (28/30) | 93% (28/30) |
| G > 7 (n=30) | 87% (26/30) | 83% (25/30) | 93% (28/30) | 97% (29/30) |
| Total positive CaP | 74% (89/120) | 78% (94/120) | 88% (106/120) | 92% (110/120) |
