# Supplementary material for: Co-expression of CD147 (EMMPRIN), CD44v3-10, MDR1 and monocarboxylate transporters is associated with prostate cancer drug resistance and progression
Source: Br J Cancer. 2010 Aug 24;103(7):1008–18. doi: 10.1038/sj.bjc.6605839 (PMC2965856; doi:10.1038/sj.bjc.6605839)
Supplement: Supplementary Table4 [file 6605839x5.doc]

**Table 4s.** CD44v3-10, MDR1, MCT1 and MCT4 immunoreactivity in primary CaP tissues (n=120)

**Specimens Immunostaining^a^ %Pos^b^ % HEG^c^**

Grade 0 1 2 3

Antigens

CD44v3-10 31 14 40 35 74% (89/120) 84% (75/89)

MDR1 26 16 41 37 78% (94/120) 83% (78/94)

MCT1 14 20 39 47 88% (106/120) 81% (86/106)

MCT4 10 22 42 46 92% (110/120) 80% (88/110)

a. Immunostaining Grade: 0= negative; 1 = weak; 2 = moderate; 3 =: strong.

b: % immunopositive tumours (Grade 1 to 3) in total of CaP samples

(Low Expression Group: LEG).

c: % tumours ≥ Grade 2 immunostaining in total of positive CaP samples

(High Expression Group: HEG).
